# Morphological and molecular description of a novel species of* Eimeria* (Apicomplexa) that infects extraintestinal tissues of kiwi (Aves: *Apteryx* spp.)

**DOI:** 10.1007/s11230-025-10227-x

**Published:** 2025-04-09

**Authors:** Emma Scheltema, Kerri Morgan, Preet Singh, Barbara Adlington, Laryssa Howe

**Affiliations:** 1 School of Veterinary Sciences, Massey University, Palmerston North, New Zealand; 2 Wildbase, Massey University, Palmerston North, New Zealand

## Abstract

Coccidia (Apiconmplexa) are naturally occurring and occasionally detrimental parasites of kiwi (*Apteryx* spp.), a unique, flightless bird species dependent upon conservation efforts for survival. Using morphological and molecular data, a new coccidia species, ***Eimeria koka* n. sp.**, isolated from two closely related but geographically isolated kiwi host species, *Apteryx rowi* Tennyson et al. (rowi) and *Apteryx mantelli* Bartlett (North Island brown kiwi), is described. Oocysts are oval (20.8 × 15.9 μm) with a mean L/W ratio of 1.3, and a distinctive rough, crenellated brown oocyst wall (mean 1.2 μm), an oocyst residuum, 1–2 polar granules, and no micropyle. Sporocysts are ellipsoidal (11.6 × 6.3 μm) with a Stieda body and sporocyst residuum. Phylogenetic analysis of the cytochrome C oxidase subunit 1 (CO1) placed ***E. koka* n. sp.** in a separate clade to other *Eimeria* species previously identified from kiwi (Coker et al., Syst Parasitol 100(3):269–281, 2023). Comparison of DNA from oocysts with infected tissues from a single juvenile North Island brown kiwi confirmed parasitism of the kidney and lung tissues. This is the first *Eimeria* species identified from extraintestinal tissues in kiwi. Further molecular studies are recommended to determine the tissue distribution of *E. koka* **n. sp.** and other *Eimeria* species in kiwi.

**Supplementary Information:**

The online version contains supplementary material available at 10.1007/s11230-025-10227-x.

## Introduction

Kiwi, the smallest extant species of ratite, are nocturnal, flightless ground-dwelling birds with an unusual burrow-nesting behaviour (Calder, 1978; Taborsky & Taborsky, 1995; Sales, 2005; Peat, 2006). There are five recognised species of kiwi (Apterygiformes: Apterygidae) in two morphological groups, all endemic to New Zealand: brown kiwi consisting of North Island brown kiwi (*Apteryx mantelli* Bartlett), rowi (*Apteryx rowi* Tennyson et al.), and tokoeka (*Apteryx australis* Shaw), and spotted kiwi consisting of little-spotted (*Apteryx owenii* Gould) and great-spotted kiwi (*Apteryx haastii* Potts) (Burbidge et al., 2003; Shepherd et al., 2012).

Kiwi populations have declined dramatically over the last century as a result of escalating anthropogenic impacts upon the natural environment, including habitat modification and successive introductions of mammalian predators (McLennan et al., 1996, 2004). Since the early 1990s, with the launch of the Kiwi Recovery Plan, there has been a significant investment in conservation interventions, such as captive-rearing (Operation Nest Egg or ONE), to maintain and, in some cases, increase kiwi populations (Butler & McLennan, 1991; Colbourne et al., 2005; Robertson et al., 2011; Germano et al., 2018). However, Coccidia (Apicomplexa), which are ubiquitous in captive and wild kiwi populations, are considered a major limiting factor in the success of captive-rearing programmes, causing morbidity and mortality in juvenile captive birds (Boardman, 2008; Morgan et al., 2014).

Coccidiosis in kiwi was first recorded in a 19-day-old captive-reared brown kiwi chick suffering from renal infection in 1978 (Thompson & Wright, 1978). Four coccidia morphotypes were reported from faeces of other kiwi chicks from the same facility, but they were unable to be sporulated or further described (Thompson & Wright, 1978). The first in-depth epidemiological, morphological, and molecular study of coccidia in kiwi was carried out by Morgan (2013). This study included a thorough analysis of sporulated oocysts from North Island brown kiwi, and the author was able to confirm that the species of coccidia routinely recovered from kiwi were of the genus *Eimeria*. Further, Morgan described four distinct species; *Eimeria apteryxii* (Morgan et al., 2017), *E. kiwii* (Morgan et al., 2017), *E. paraurii* (Morgan et al., 2017), and *E. mantellii* (Morgan et al., 2017) from North Island brown kiwi. Subsequently, one additional species from North Island brown kiwi*, Eimeria paopaoii* (Coker et al., 2023), has been described. Infection with multiple species of *Eimeria* is common in kiwi, and while infection typically occurs in the intestine, it occasionally occurs in extraintestinal tissues, including the kidney, liver, lungs, and spleen (Morgan et al., 2012, 2013). However, which *Eimeria* species cause these differing disease pathologies is currently unknown.

These previous studies have described coccidia recovered from a relatively small number of individual North Island brown kiwi from a limited number of geographic locations. Although most *Eimeria* species are highly host specific, recent research shows that some have a wider host spectrum than traditionally thought; several *Eimeria* species are shared across multiple host genera or family groups (Duszynski & Wilber, 1997; Mácová et al., 2018; Kvičerová et al., 2020; Trefancová et al., 2021). Coker (2021) reported *Eimeria* species from Haast tokoeka (*A. australis* “Haast”) that were morphologically synonymous with four of the species described to date from North Island brown kiwi. Given that there has been limited investigation of coccidia infecting other species of kiwi across a variety of geographic locations, it is possible that more kiwi *Eimeria* species have yet to be identified and described. In addition, translocations of kiwi for the purposes of conservation inadvertently bring together hosts and parasites that are normally geographically isolated, and may have facilitated parasite transmission between members of the genus *Apteryx* (Morgan et al., 2017; Coker, 2021; Jahn et al., 2022).

In the present study, a morphologically distinct species of *Eimeria* recovered from both rowi and North Island brown kiwi, from multiple geographic locations around New Zealand, is described. Establishing basic biology and taxonomy of *Eimeria* species in kiwi is a fundamental step in developing tools to detect and manage disease.

## Materials and methods

### Morphological analysis

#### Faecal sample collection and storage

Coccidia-positive faecal samples were received from routine diagnostic screening of kiwi carried out at Massey University’s Parasitology Department (Massey University, Manawatū, New Zealand). Samples were submitted from captive and creche (protected wild) locations around New Zealand to the laboratory, usually within 24 hours of collection, and stored in sterile plastic containers at room temperature until analysis (≤ 2 days). Samples were determined to be coccidia positive via centrifugal flotation (CFF) procedure (Ministry of Agriculture Fisheries and Food [MAFF], 1986). Positive samples were further screened using the mini-FLOTAC method for more accurate oocyst quantification (Cringoli et al., 2017; Coker et al., 2020). Any samples that were observed to have oocysts of the novel species were set up for sporulation and further morphological and molecular analysis as described below.

#### Sporulation

Approximately 0.25 g of sample was mixed with a 2% (w/V) aqueous potassium dichromate (K_2_Cr_2_O_7_) solution in a thin layer (~3–5mm) within petri dishes, placed within a sealed plastic box to prevent desiccation, and maintained at room temperature. Samples were screened regularly (every 2–3 days) until oocyst sporulation was detected.

#### Oocyst measurement

Once sporulated, a modified faecal flotation was carried out to isolate oocysts for measurement and imaging (Coker, 2021). Briefly, approximately 0.5–1 mL of faeces-potassium dichromate homogenate was aliquoted using a 3mL pipette into a 1.7 mL Eppendorf tube and volume made up with distilled water to 1.7 mL. The sample was centrifuged at 13,000 rpm (12,470 g) for 4 min to concentrate oocysts to the bottom of the tube. The excess solution was carefully aliquoted off so as not to disturb the pelleted sample and discarded. Saturated magnesium sulfate solution (MgSO_4_, SG 1.28) was added to the tube, the sample mixed well, and then centrifuged at 5000 rpm (1844 g) for 5 min to float oocysts. An approximately 500 μL aliquot of the oocyst-salt solution was collected from the surface of the solution with a micropipette and deposited into a custom glass McMaster-style slide. The oocysts were allowed to float to the surface for one minute, after which the sample was examined for floated oocysts under the microscope and images taken.

Imaging and measurements were carried out using an Olympus BH-2 (Olympus Optical Co. Ltd., Tokyo, Japan) microscope using a Canon Digital Rebel xTi DSLR (Canon Inc., Tokyo, Japan) with C-mount adapter. Images were taken digitally under oil immersion at a magnification of 1000×. Morphological features were measured from 100 oocysts (except sporocysts—see Morgan et al., 2017) using ImageJ, ver. 1.53 q (Rasband, 1997). For each oocyst, up to three images were measured for both oocyst and sporocyst length and width with the maximum measure from the replicates used for calculation of averages. Measures of oocyst wall width and other structures (polar granule, Stieda body) were taken as averages across all measures. Up to three sporocysts were measured for each oocyst, depending on their orientation. Oocysts at an angle to the photographic plane were omitted, which resulted in no appropriate sporocysts for that oocyst in some cases.

#### Morphometric analysis

Statistical analysis of oocyst morphometrics was carried out using R (R Core Team, 2023). Mean, standard deviation, and size range were calculated for each morphological parameter. Length:width (L/W) ratio data from the first 100 measured oocysts of the new *Eimeria* species were plotted alongside L/W measurements of the five previously described species of kiwi *Eimeria* (Coker, 2021; Morgan, 2013) to visualise shape differences. Scatterplots were made with the addition of measurements used to characterise the five previously described kiwi *Eimeria* species (Morgan, 2013; Coker, 2021). The difference in L/W ratios between all species was tested for significance using ANOVA with a linear model (species as a predictor of L:W ratio) and Tukey’s HSD test to allow for multiple comparisons of means, with 95% family-wise confidence level and significance level of p < 0.05.

#### Illustrations

Phototype images were taken on an Olympus IX83 microscope with DIC optics using a 100× (NA1.4) objective lens. Images were captured with a Retiga 6000 monochrome camera (QImaging) controlled by cellSens Dimension software (v1.18; Olympus). Additional images were captured using a DM750 light microscope and ICC50W microscope camera (Leica Microsystems, Wetzlar, Germany). Histological images were focus-stacked using Picolay software (Cypionka, 2024). Oocyst composite line drawings were hand drawn and edited with Adobe Photoshop CS5 (Adobe Systems, San Jose, CA, USA).

### Molecular analysis

#### DNA extraction

Total DNA from faecal samples containing oocysts of *E. koka* **n. sp.**, as morphologically identified on standard faecal screening, was extracted to obtain a molecular description of species. Additionally, DNA from representative tissues from intestine, liver, kidney, lung, and spleen from a juvenile kiwi that had died as a result of coccidiosis was also extracted and analysed molecularly to determine the *Eimeria* species causing infection.

Subsamples of faeces containing the new species (n = 2) identified through routine screening were frozen at − 80 °C for >24 hr. DNA was extracted from 0.15 g of each sample using the Quick-DNA Faecal/Soil Microbe DNA Miniprep extraction kit (ZYMO Research, Orange County, CA, USA) following the manufacturer’s instructions with modifications (boiling and freezing, followed by overnight proteinase-K digestion at 56 °C) as described by Coker (2021) to disrupt the tough oocyst wall. Extracted DNA was eluted into 70 uL elution buffer. Negative controls (water) were used in each extraction group to check for contamination. Extracted DNA was tested on a Nanodrop™ 2000 spectrophotometer (Thermofisher Scientific, Waltham, MA, USA) to measure DNA concentration and quality and then stored at − 20 °C until analysis.

Formalin-fixed paraffin-embedded (FFPE) tissue blocks of a range of tissues known to be infected with coccidia (intestine, kidney, liver, lung, spleen) from a deceased kiwi chick were collected from the Massey University School of Veterinary Science’s pathology collection for retrospective molecular analysis.

A subsample of each FFPE tissue (10 μm) was prepared for PCR analysis, flanked by two thinner samples (4 μm) for Haematoxylin & Eosin (H&E) staining for histological analysis, using a microtome with a fresh blade between samples. Each 10 μm slice was stored in individual Eppendorf tubes until DNA extraction. Where more than one type of tissue, such as liver and kidney, was present on a single FFPE block, the paraffin block was melted, and the target tissue was carefully removed manually with forceps and prepared in a new paraffin-embedded cassette before samples were then collected as described for individual tissue DNA extraction.

DNA extraction was performed on 10 μm scrolls of FFPE tissues using the manufacturer guidelines for a commercially available kit (Qiagen DNeasy® Blood and Tissue kit, Valencia, CA, USA). DNA was eluted using only one elution of 70 μL of the provided elution buffer to concentrate the eluted DNA. Extracted DNA was tested on a Nanodrop™ 2000 spectrophotometer (Thermofisher Scientific, Waltham, MA, USA) to measure DNA concentration and quality. DNA samples were stored at − 20 °C until PCR analysis was performed.

#### PCR amplification of CO1 gene

Conventional PCR was carried out targeting a 220 bp region of the *Eimeria* CO1 gene using CokerF2 (Coker, 2021)/CO1R2 (Yang et al., 2013) primers and PCR reaction conditions as developed by Coker (2021) (Table 1). Briefly, 1X PCR buffer, 1.5mM MgCl_2_, 200 nM each of dNTPs (Invitrogen, ThermoFisher Scientific, Waltham, MA, USA), forward and reverse primers, 5U Platinum™ Taq DNA polymerase (Invitrogen), and 10–70 ng/rxn DNA were combined for a total reaction volume of 20 μL. Cycling conditions were: initial denaturation 94 °C for 2 min, then 40 cycles of denaturation at 94 °C for 20 sec, annealing at 55 °C for 30 sec, and extension at 72 °C for 30 sec with a final extension of 72 °C for 10 min.

**Table 1:** Primers for amplification of partial mitochondrial cytochrome c oxidase (CO1) genes from *Eimeria* spp. oocysts recovered from the faeces of North Island brown kiwi (*Apteryx mantelli*)

Primer/probe name	Sequence (5’–3’)	Target sequence length (bp)	Target	References
CokerF2	5’ AYG ATG CYT CYT TTA ATG GTG A 3’	220	***Eimeria* spp.**	Coker (2021)
CO1R2	5’ GTC ATC ATA TGR TGT GCC CA 3’			Yang et al. (2013)

#### Sequence analysis

PCR products were visualised on 1.5% w/v agarose gels (Invitrogen UltraPure Agarose, ThermoFisher Scientific, Waltham, MA, USA), using RedSafe Nucleic Acid Staining Solution (iNtRON Biotechnology, Gyeonggi-do, South Korea). Samples with bands of the correct size (220 bp) were purified for sequencing as follows. The positive PCR products were excised from the agarose gel and frozen overnight at − 20 °C before using a homemade spin-column protocol (Sun et al., 2012) to purify the PCR products. All purified products were sent for forward and reverse Sanger sequencing at Massey Genome Service (Massey University, Palmerston North, New Zealand). Sequences were analysed in Geneious Prime v. 11.0.14.1+1 (Biomatters, Auckland, New Zealand).

#### CO1 phylogenetic analysis

A phylogenetic tree was constructed for the newly identified kiwi *Eimeria* species at the CO1 loci using an additional 37 sequences with 81–91% homology, gathered from BLASTn (v. 2.16.0+) (Altschul et al., 1990; Zhang et al., 2000) search of NCBI GenBank database, plus a DNA sequence extracted from renal tissue from an infected North Island brown kiwi. *Toxoplasma gondii* (NCBI: JX473253) was used as an outgroup. Sequences were aligned using MUSCLE (v. 3.8.425) (Edgar, 2004) in Geneious Prime v. 11.0.14.1+1 (Biomatters) and trimmed to the same length.

Bayesian posterior probabilities generated in MrBayes (v.3.1.2) (Ronquist et al., 2012) using Markov-chain Monte Carlo (MCMC) methods was carried out on the resulting alignment to determine likely evolutionary lineage of ***Eimeria koka*** **n. sp.** The Bayesian phylogeny was obtained using one cold and three hot Monte Carlo Markov chains, which were sampled every 1000 generations over two million generations; 2000 trees were generated. Of these trees, 25% were discarded as burn-in material. The remaining trees were used to construct a majority consensus tree.

The sequence divergence between and within the different lineages was calculated using a Jukes–Cantor model of substitution implemented in the programme PAUP* 4.0 Beta version 10 (Swofford, 2002).

Unique DNA sequences from oocysts of the new species of *Eimeria* (21021) and DNA extracted from infected kidney (51506) were deposited in GenBank under the reference numbers PQ283206 and PQ285481.

### Histology

Histological assessment was carried out on new H&E slides prepared from archived formalin-fixed paraffin embedded (FFPE) tissues. Both H&E slides taken on either side of the FFPE sample for DNA extraction were thoroughly examined under light microscopy at 100–400× magnification for the presence of intracellular coccidial stages.

## Results

During the 2021–2022 kiwi breeding season, 70 coccidia-positive faecal samples were screened from North Island brown kiwi (59/70) and rowi (11/70). ***Eimeria koka* n. sp.** was observed in 6/70 samples (9%). Demographic details of kiwi from which samples positive for the new species are summarised in Table 2.

**Table 2:** Kiwi (*Apteryx* spp.) faecal samples with positive detections of ***Eimeria koka* n. sp.** collected from various captive and creche locations during the 2021–2022 breeding season

Sample #	Host species	Host age	Facility	Location	Collection date	Other species present
21021	*Apteryx rowi*	7 weeks	Willowbank Wildlife Reserve	Christchurch	24/11/2021	*E. kiwii*
21020	*Apteryx rowi*	7 weeks	Willowbank Wildlife Reserve	Christchurch	23/11/2021	*E. kiwii*
21560	*Apteryx mantelli*	6 months	National Aquarium	Napier	28/03/2022	*E. kiwii* *E. apteryxii* *E. mantellii*
21550	*Apteryx mantelli*	5 ½ months	Warrenheip Creche	Cambridge	28/03/2022	*E. kiwii* *E. apteryxii* *E. paraurii*
21537	*Apteryx mantelli*	6 months	Butterfly Creek	Auckland	21/03/2022	*E. kiwii*
21631	*Apteryx mantelli*	1 year	Nga Manu Nature Reserve	Waikanae	11/04/2022	*E. kiwii* *E. mantellii*

The first detection was in samples from rowi, and this sample (21021) was sporulated and used to describe the species as *Eimeria koka* **n. sp.**

## *Eimeria koka *n. sp*.*

Family: Eimeriidae Minchin, 1903

Genus**:** *Eimeria* Schneider, 1875

**Fig. 1:**
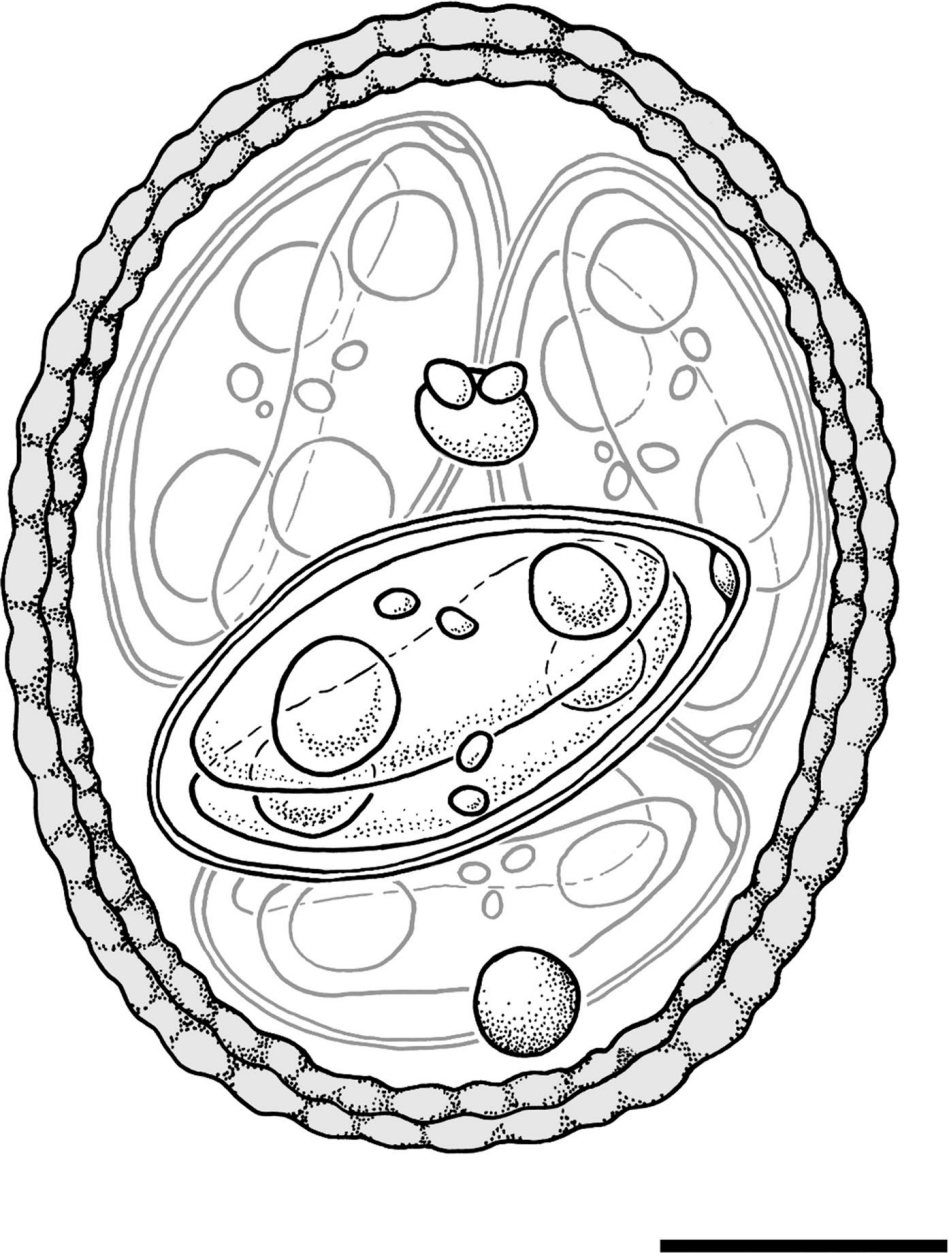
Composite line drawing of a sporulated oocyst of *Eimeria koka* **n. sp.** isolated from rowi kiwi (*Apteryx rowi* Tennyson et al.). Scale bar = 5 μm.

Isolate name: ***Eimeria koka* n. sp.** (Fig. 1)

Type host: *Apteryx rowi* Tennyson et al. (rowi) ; seven weeks old (chick).

Other species: *Apteryx mantelli* Bartlett (North Island brown kiwi); five to 12 months old (chick to subadult).

Localities: Butterfly Creek, Auckland (− 36.99789, 174.79412); National Aquarium, Napier (− 39.47957, 176.87609); Nga Manu Nature Reserve, Waikanae (− 40.86032, 175.05938); Warrenheip Creche, Cambridge (− 37.90572, 175.55363); Willowbank Wildlife Reserve, Christchurch, New Zealand (− 43.46353, 172.59399).

Deposited material: Preserved oocysts specimens and photosyntype are deposited in the Te Papa Tongarewa collection under reference: PR.000004. Representative DNA sequences have been deposited in GenBank under the accession numbers PQ283206 and PQ285481.

Zoobank registration: *Eimeria koka* has been registered with Zoobank with the Life Science Identifier urn:lsid:zoobank.org:act:FCCB9CA9-623D-4F25-A863-037794AAC6BD.

Prevalence: 9% (in 6/70 screened samples). In this study, ***E. koka* n. sp.** was detected via faecal flotation in 4/59 (7%) faecal samples from North Island brown kiwi and 2/11(18%) samples from rowi. It is likely that these numbers do not represent the true prevalence of this species in the wild kiwi population as these samples were obtained from captive-reared birds. The overall presence in the host kiwi population is unknown.

Sporulation: Exogenous. All oocysts were passed unsporulated and sporulated within 10 days at room temperature.

Site of infection: Extraintestinal. The sequence obtained from faecal sample (21021) matched sequences obtained from kidney tissue screened from a single pathology case in this study but did not match sequences obtained from infected intestinal tissue. Because tissues were only analysed from one bird, it was impossible to identify the primary infection site or to rule out intestinal infection by this species.

Sporulated oocysts: Sporulated oocysts (n = 100), large and ellipsoidal, 20.8 μm (17.7–23.8 μm) length by 15.9 μm (14.1–17.6 μm) wide; length-width (L/W) ratio 1.3 μm (1.13–1.53 μm). Oocyst wall brown-coloured, bilayered, 1.20 μm (0.99–1.47 μm) thick, with a rough, crenellated surface. Micropyle absent, compact circular oocyst residuum present, and usually 1–2 polar granules (1.81–2.02 μm diameter) present.

Sporocysts: Sporocysts (n = 103) 4, ellipsoidal, 11.6 μm (9.9–12.9 μm) long × 6.3 μm (5.1 × 7.6 μm) wide; L–W ratio 1.9 (1.5–2.4). Stieda body present, flattened oval c. 1 μm deep × 1.7 μm wide; sub-Stieda and para-Stieda bodies absent; sporocyst residuum present, consisting of several scattered spheres among sporozoites.

Sporozoites: Two sporozoites elongated ellipsoid in shape. Not measured. Sporozoite nucleus not observable. Anterior and posterior refractile bodies present.

Prepatent period: Unknown

Patent period: Unknown

Etymology: “Koka” is a Māori term for a rough cape made of undressed leaves (such as harakeke) and the colour brown (stative). Māori is the indigenous language of New Zealand. This descriptor was chosen in reference to the rough brown oocyst wall of this species of *Eimeria*, which is its distinguishing characteristic from other described kiwi *Eimeria* species.

**Fig. 2:**
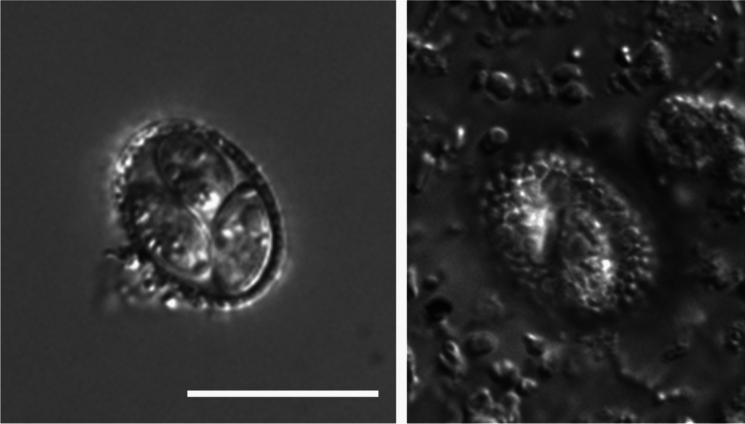
Novel species, *Eimeria koka* **n. sp.**, isolated from rowi (*Apteryx rowi* Tennyson et al.) imaged using differential interference contrast; **A** internal structure showing three of the four ellipsoidal sporocysts, **B** outer oocyst wall, illustrating its unique rough surface texture. Scale bar = 20 μm.

**Fig. 3:**
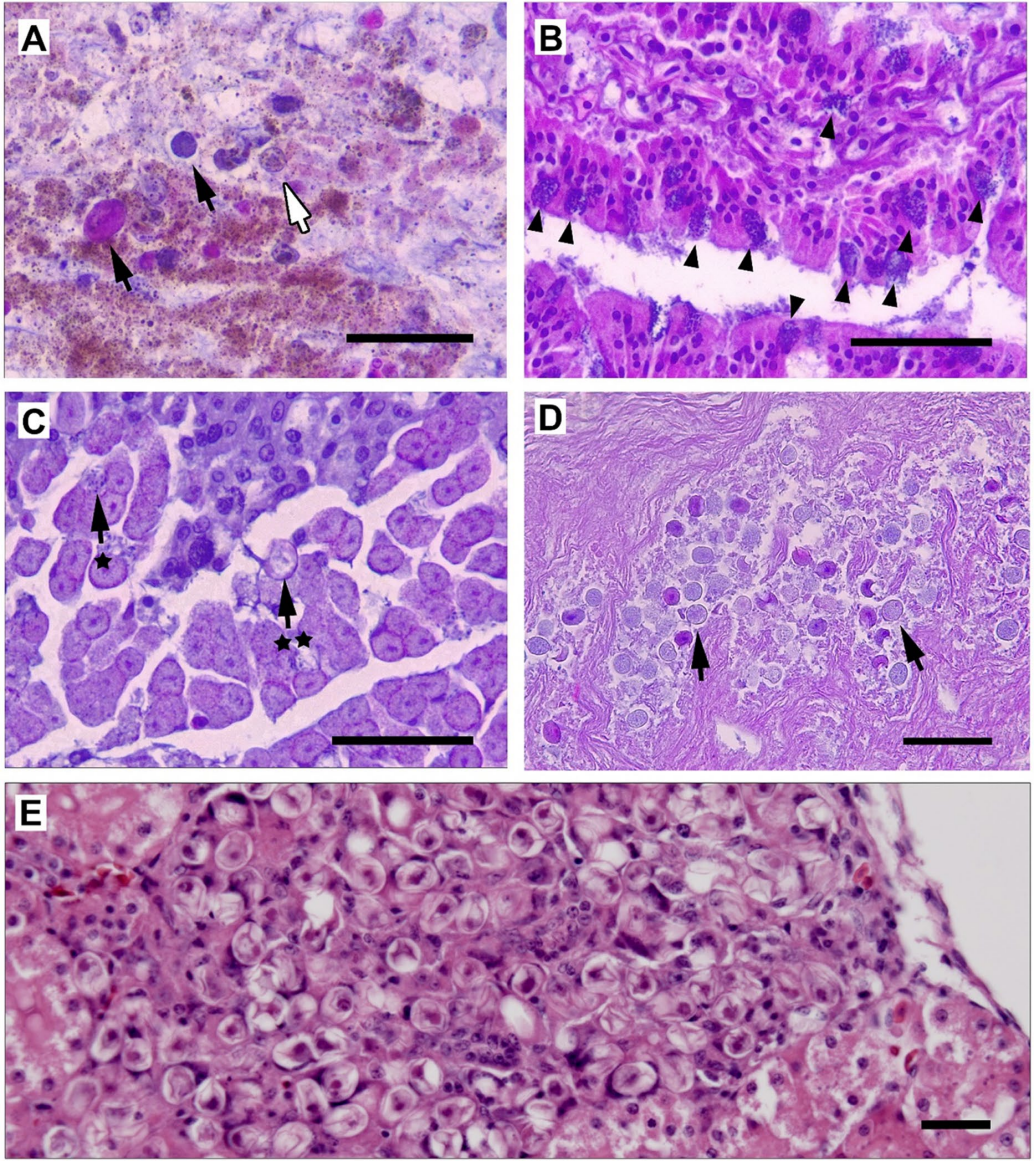
Coccidial stages observed in H&E slides from a seven-week-old juvenile North Island brown kiwi (*Apteryx mantelli*) that died with severe intestinal and renal coccidiosis; **A** oocysts (black arrows) and gametocytes (white arrow) of several different morphologies amongst intestinal contents and sloughed cells (scale bar = 50 μm); **B** microgametocytes, with flagellate microgametes, in epithelial cells (indicated) lining the crypts of the intestine (scale = 50 μm); **C** close up of microgametocyte (*) and early oocyst (**) amongst many gametocytes, in epithelial cells within the branch of the ureter (scale = 50 μm); **D** close up of gametocytes and early oocysts within the lumen of the gall bladder (scale = 50 μm); **E** early oocysts, of similar dimensions to exogenous oocyst form, observed forming in renal tubule epithelial cells (scale = 25 μm).

Taxonomic remarks:

***Eimeria koka* n. sp.** is morphologically distinct from *Eimeria* species described from kiwi to date with its distinctive brown colour and thick, crenellated outer oocyst wall (Figs. 1, 2). Of the described species, ***E. koka* n. sp.** appears most similar to *E. paraurii* described by Morgan et al. (2017), but ***E. koka* n. sp.** is considerably smaller in size. This morphotype was detected during routine faecal sampling and was observed in two species of kiwi across multiple geographic locations (Table 2). While it is easily distinguished because of its unusual morphology, it is typically found at low prevalence within mixed-species samples, and so it is possible that it is missed on standard diagnostic screening. This species is suspected to replicate within the kidneys (as early oocysts with equivalent dimensions to the exogenous oocysts were observed on renal histopathology but no other extraintestinal organs) (Fig. 3E). The presence of this coccidian in multiple samples across different geographic regions, the confirmation of infection within post-mortem tissue samples, and the viability of oocysts all indicate that this is a parasite of kiwi.

### Morphological analysis

#### Oocyst morphometrics

Mean (±SD) for each of the morphometric parameters were calculated for the novel species, *Eimeria koka*, and are presented in comparison to previously described kiwi *Eimeria* species (Morgan et al., 2017; Coker et al., 2023) in Table 3.

**Table 3:** Morphological and biological comparison of ***Eimeria koka* n. sp.** from a rowi kiwi (*Apteryx rowi*) with other previously described kiwi *Eimeria* species (Coker et al., 2023; Morgan et al., 2017)

Oocyst	*Eimeria kiwii*		*Eimeria apteryxii*		*Eimeria mantellii*		*Eimeria paraurii*		*Eimeria paopaoii*		*Eimeria koka* n. sp*.*			
	Spherical to subspherical		Ovoid to pyriform		Ovoid tapering to blunt narrow end		Pyriform		Circular to elliptic		Large oval, brown			
*Length*	14.8	(11.6–22.0)	23.9	(10.3–28.2)	17.8	(16.1–19.8)	32.2	(26–44)	14.6	(10.6–18.4)	20.8	±	1.2	(17.7–23.8)
*Width*	13.9	(10.6–19.0)	14.9	(12.7–20.7)	10.7	(9.6–12.0)	19.8	(16–23)	13.9	(9.8–17.2)	15.9	±	0.6	(14.1–17.6)
Shape index (L/W ratio)	1.1	(1.0–1.2)	1.6	(1.2–2.0)	1.7	(1.3–2.0)	1.6	(1.3–2.3)	1.1	(1.0–1.2)	1.3	±	0.1	(1.1–1.5)
Oocyst wall	Striated		Smooth		Smooth		Striated, rough		Smooth		Rough			
	0.8	(0.5–1.1)	0.8	(0.6–1.2)	0.6	(0.4–0.8)	1.2	(0.8–1.7)	0.7	(0.5–0.9)	1.2	±	0.1	(1.0–1.5)
Micropyle	Absent		Present, 2.0 (1.1–3.2)		Absent		Present, 4.1 (2.5–5.6)		Absent		Absent			
Oocyst residuum	Absent		Absent		Absent		Absent		Absent		Present			
Polar granule	Usually 1–2 present		Usually 1–2 present, sometimes up to 7		Usually 1, sometimes 2 present		Usually 1–2 present		1 present		Usually 1–2 present			
*Length*	2.1	(1.5–2.0)	2.2	(1.2–2.9)	1.5	(0.9–2.1)	NA		NA		2.0	±	0.2	(1.5–2.9)
*Width*	1.6	(1.0–2.1)	1.3	(0.8–2.1)	1.1	(0.4–1.6)	NA		NA		1.8	±	0.2	(1.2–2.3)
Sporocyst														
*Length*	9.4	(6.5–13.6)	11.7	(8.0–17.4)	9.5	(7.9–10)	16.2	(11.7–20.6)	9.4	(5.8–11.5)	11.6	±	0.7	(9.9–12.9)
*Width*	4.9	(3.6–7.4)	6.0	(5.0–8.3)	4.7	(3.9–5.2)	7.9	(6.8–9.2)	5.1	(3.6–6.7)	6.3	±	0.4	(5.1–7.6)
Sporocyst shape index (L/W ratio)	1.9	(1.5–2.3)	1.9	(1.4–2.4)	2.0	(1.6–2.4)	2.0	(1.5–2.3)	1.9	(1.3–2.2)	1.9	±	0.2	(1.5–2.4)
Sporocyst residuum	Present, loosely clumped, usually located between two sporozoites		Present, tightly clumped, centrally located		Present, clumped, centrally located		Present, generally clumped		Present, generally clumped		Present. Several scattered spheres			
Sporozoite	Ellipsoid-ovoid		Ellipsoid. Large refractile body		Ellipsoid		Large refractile body at base		Large refractile body at posterior end		Elongate ellipsoid. Anterior and posterior refractile bodies			
Stieda body	Present, small not obvious		Present		Present		Present		Present		Flattened oval ~1.01 μm deep × 1.7 μm wide			
Hosts	*Apteryx mantelli*		*Apteryx mantelli*		*Apteryx mantelli*		*Apteryx mantelli*		*Apteryx mantelli*		*Apteryx rowi, Apteryx mantelli*			
Type locality	Maungatautari ecological island, Waikato and Pukaha, Mt Bruce, Waiarapa, New Zealand		Maungatautari ecological island, Waikato and Pukaha, Mt Bruce, Waiarapa, New Zealand		Maungatautari ecological island, Waikato		Pukaha Mount Bruce, Wairarapa, New Zealand		Wairarapa; Waikato; Rotorua, New Zealand		Christchurch; Napier; Cambridge; Auckland and Waikanae, New Zealand			
Reference	Morgan et al. (2017)		Morgan et al. (2017)		Morgan et al. (2017)		Morgan et al. (2017)		Coker et al. (2023)		This study			

Measurements are in μm with the mean followed by the range (in parentheses). Mean±SD are provided for *E. koka* **n. sp.**

*NA* not available

**Fig. 4:**
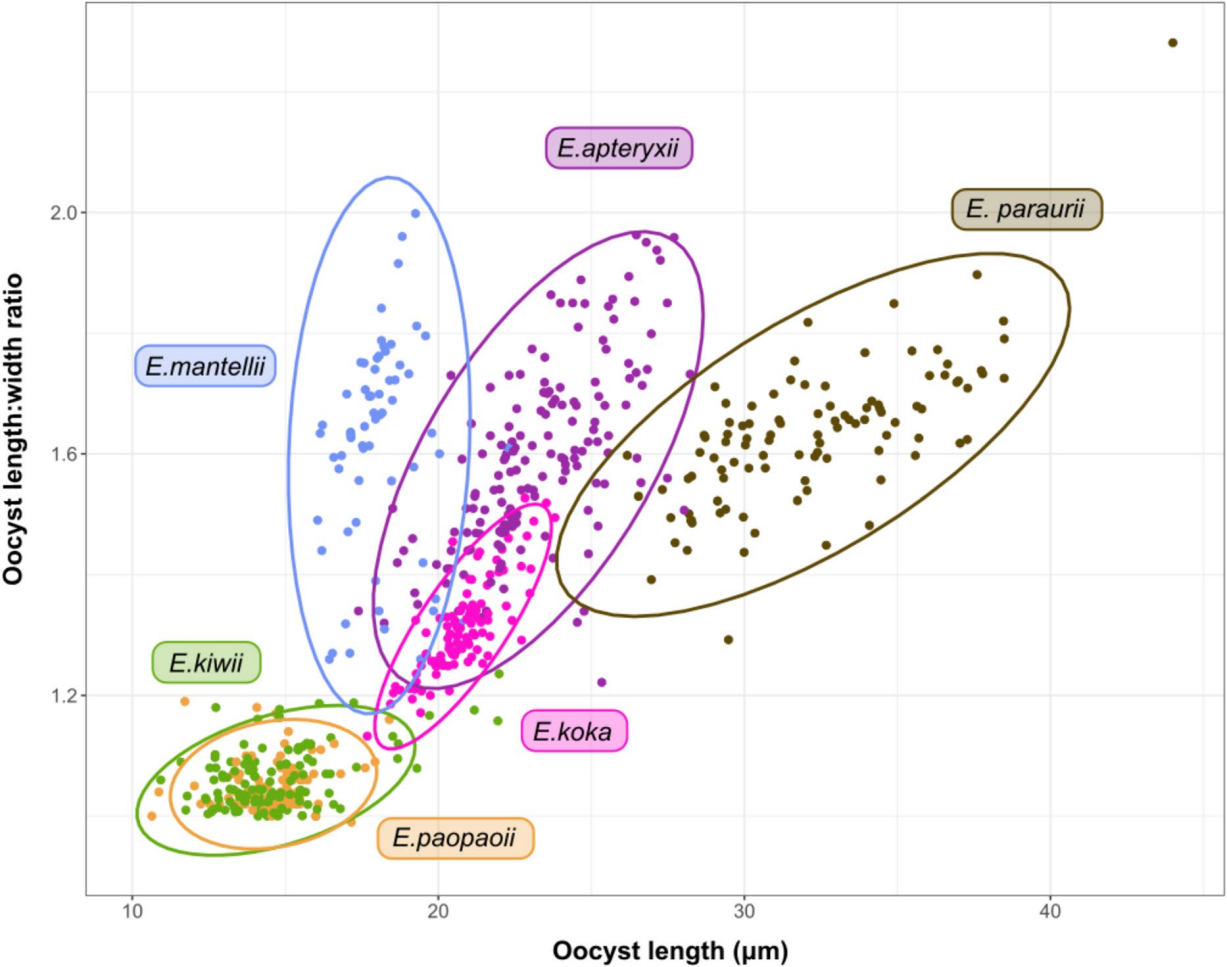
Comparison of oocyst length and length:width ratio (L/W ratio) from ***Eimeria koka* n. sp.** from this study (pink) and previously described *Eimeria* spp. from North Island brown kiwi (*Apteryx mantelli*) (data Morgan (2013) and Coker (2021)). Ellipses represent 95% confidence intervals of oocyst shape based on observed length:width and length measurements, for each species.

**Fig. 5:**
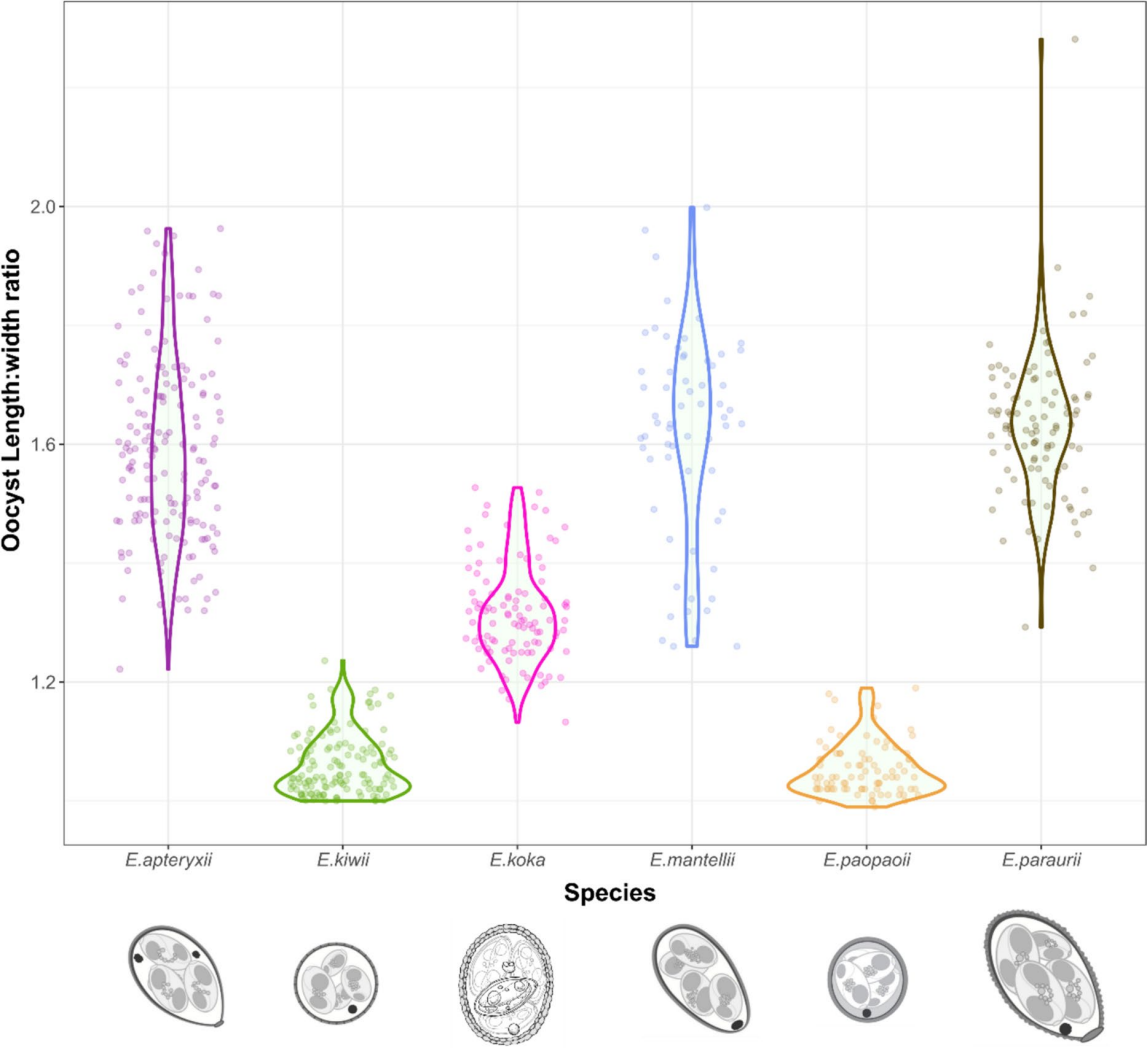
Violin (density) plot of oocyst length:width ratio (L/W ratio) of ***Eimeria koka* n. sp.** from this study (pink) and previously described *Eimeria* spp. from North Island brown kiwi (*Apteryx mantelli*) (data from Morgan (2013) and Coker (2021)). Oocyst images from Morgan et al. (2017) and this study.

Comparison of shape index (length:width ratio) for *E. koka* with previously described species demonstrates clustering of data points, most closely clustering with *E. apteryxii* and *E. mantelli* (Morgan et al., 2017). This novel morphotype differs from these two described species in that it is, on average, larger than *E. mantelli* and has a smaller L-W ratio than both *E. apteryxii* and *E. mantellii.* There appears to be less observed size variation in *E. koka* than *E. mantelli*, *E. apteryxii,* or *E. paraurii* (Figs. 4, 5).

Comparison of oocyst length-width ratios between species indicates that *E. koka* significantly differs in shape to *Eimeria apteryxii*, *E. kiwii*, *E. mantellii*, *E. paopaoii*, and *E. paraurii* (all p < 0.001). The only species that did not significantly differ in L:W ratio were *E. mantellii* and *E. paraurii* (p = 0.93), *E. mantellii* and *E. apteryxii* (p = 0.72), and *E. kiwii* and *E. paopaoii* (p = 0.999) (Tukey HSD, p < 0.05).****

### Molecular analysis

#### Faecal samples

A 220 bp region of mt CO1 was successfully sequenced from the original single-species faecal sample (21021) from rowi using CokerF2 (Coker, 2021)/CO1R2 (Yang et al., 2013) primers. Attempts to extract and sequence clean DNA from additional faecal samples were hindered by the presence of multiple *Eimeria* species (Table 2). BLAST analysis of the novel *E. koka* (GenBank PQ283206) sequence revealed it shared 91.4% identity with an *Eimeria* species recovered from *Chloroceryle americana* (GenBank OL773690); 90.9% identity with *Eimeria potoroi* recovered from *Potorus tridactylus* or long-nosed potoroo (GenBank MK202807); and 90.4% identity with an *Eimeria* species from *Suncus murinus* or Asian house shrew (GenBank MN184724).

#### Tissue samples

A 220 bp region of CO1 DNA was successfully amplified from kidney, liver, lung and intestinal tissue from a seven-week-old North Island brown kiwi (*A. mantelli*) juvenile who died because of severe renal and intestinal coccidiosis (Massey University School of Veterinary Science’s pathology collection^1^). There was no PCR amplification from spleen tissue. DNA sequences were successfully obtained from kidney (GenBank PQ285481) and intestinal tissue. The CO1 DNA sequence obtained from the *E. koka* type faecal sample was a 100% sequence homology to the sequence obtained from infected kidney tissue. There was no match between *E. koka* type faecal DNA and that sequenced from coccidia-infected intestinal tissue, which appeared to represent a mixed-species infection on sequencing (due to presence of multiple peaks).

#### CO1 phylogenetic analysis

**Fig. 6:**
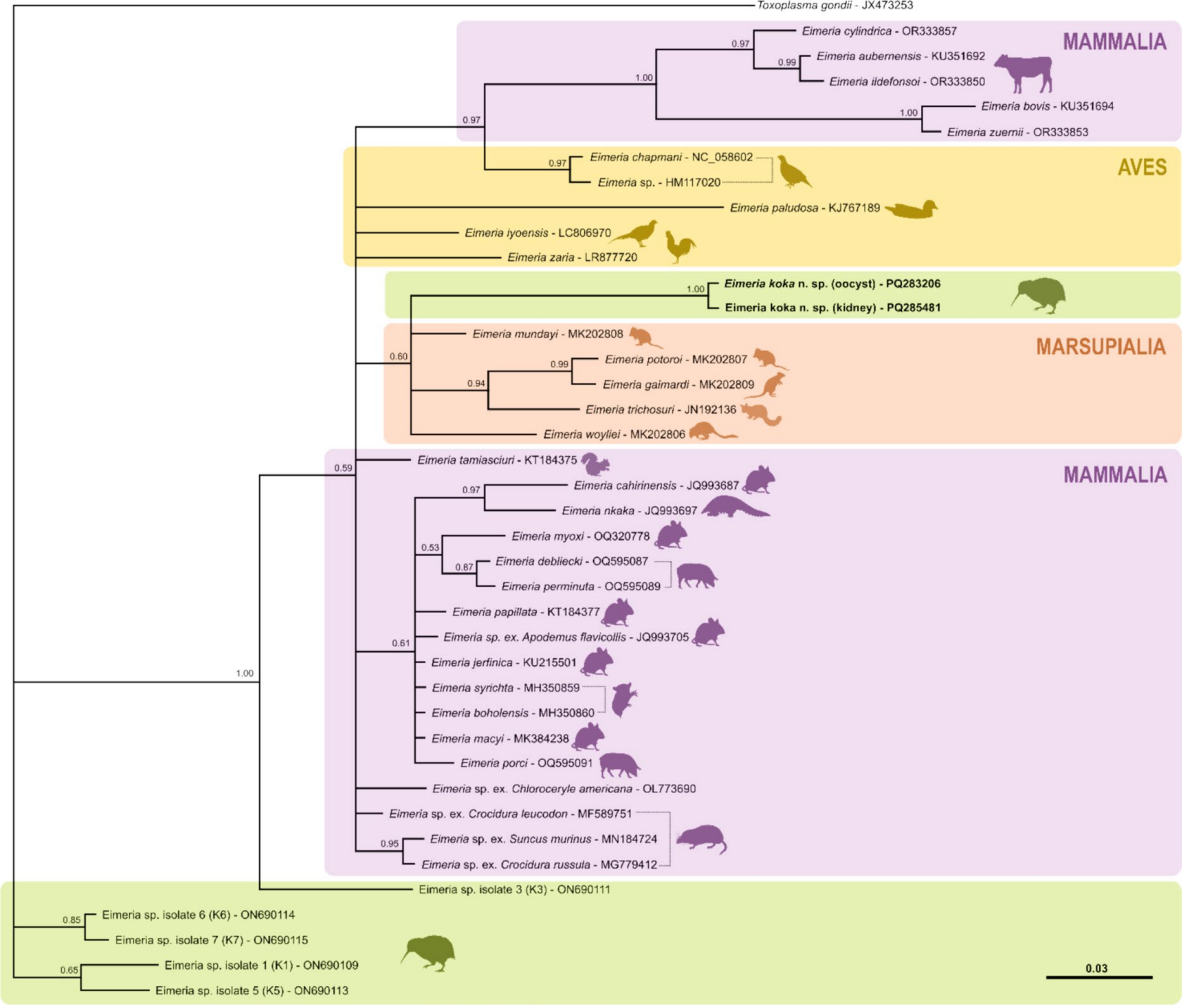
Bayesian phylogenetic analysis and comparison of a 210 bp region of mitochondrial cytochrome b gene from *Eimeria koka* **n. sp.** isolates obtained from faecal oocysts from North Island brown kiwi (*Apteryx mantelli*) and kidneys from rowi (*Apteryx rowi*) (in bold) with 37 previously published *Eimeria* spp. sequences present in the GenBank database. *Eimeria* spp. isolates from kiwi from this study, and four previously identified kiwi *Eimeria* genotypes (Coker et al., 2023), are indicated in green boxes. *Toxoplasma gondii* is used as an outgroup. Branch lengths are drawn proportionally to the amount of genetic change (substitutions per site). Posterior probability values are indicated above branch nodes. Genbank accession numbers of the sequences are given after the species names of the parasites.

Phylogenetic analysis of the *Eimeria koka* CO1 sequences obtained from both the faecal and kidney tissue samples grouped within a clade that includes Australian potoroid marsupials (Fig. 6): *Eimeria woyliei* (from *Bettongia penicillata* or woylie) (89.5% sequence homology); *Eimeria mundayi* (89.5% sequence homology) and *Eimeria potoroid* (from *Potorus tridactylus* or long-nosed potoroo) (90.9% sequence homology); *Eimeria gaimardi* (from *Bettongia gaimardi* or Eastern bettong) (90% sequence homology); and *Eimeria trichosuri* (from *Trichosurus caninus* or mountain brushtail possum) (88.5% sequence homology) (Northover et al., 2019a).

*Eimeria koka* formed a distinct clade compared to *Eimeria* isolates previously recovered and described from brown kiwi (Coker et al., 2023) (Fig. 6). *E. koka* shared 83–88% similarity with these other kiwi *Eimeria* isolates, which consist of mixed-species samples representative of the five species previously described from North Island brown kiwi (Morgan et al., 2017; Coker et al., 2023).

### Histology

H&E slides were reviewed of intestine, liver, kidney, spleen, and lung from the seven-week-old kiwi (Fig. 3). Some of the observed tissues were partially autolysed. Coccidial organisms were observed in intestine (meronts, gametocytes, oocysts) and kidney tissue (meronts, gametocytes, and oocysts), including within the epithelial cells of a branch of the ureter (Fig. 3D). Several distinct morphotypes of gametocytes and oocysts were observed in the intestinal contents, suggestive of a multi-species infection (Fig. 3A). Only one morphotype of sexual stages was observed in the kidney and ureter (Fig. 3C, E). Early oocysts in kidney were oval in shape with mean dimensions of 16.7 × 12.8 μm (L:W ratio = 1.3) and gametocytes had mean dimensions of 15.9 × 11.3 μm (L:W ratio = 1.43).

No coccidial stages were observed in liver, lung, or spleen tissue; however, early oocyst stages (mean dimensions of 17.0 × 13.9 μm [L:W ratio = 1.2] were incidentally observed in the lumen of a section of gall bladder attached to the liver tissue on the same slide (Fig. 3D). Due to the autolysed condition of the tissue, it was not able to be determined whether these sexual stages originated from the gall bladder or were transient.

## Discussion

The newly described species is morphologically distinct from the five previously described species of kiwi *Eimeria*, primarily distinguished by its large size, as compared to the most commonly observed species (*E. kiwii* and *E. apteryxii*), oval shape and its thick, crenellated rough oocyst wall, which has a distinctive brown coloration. The most similar species in appearance is *E. paraurii* with its rough oocyst wall (Morgan et al., 2017); however, it is much larger and more ovate in shape than *E. koka*. Morgan et al. (2017) suggested that *E. paraurii* was, in fact, the “type C” coccidia observed by Thompson and Wright (1978) when they made the first record of coccidia recovered from kiwi. Based on comparable morphometrics and distinctive shape, it appears possible that *E. koka* is synonymous with oocyst “type D”, also described in the same study, thus further confirming this species distinction. *E. mantellii* was the next most morphologically similar in size and L/W ratio, but this species has a distinct teardrop shape and a thin, smooth, and colourless oocyst wall. Due to its distinctive features, *E. koka* is relatively easily differentiated on a standard faecal float in comparison with some of the previously described kiwi *Eimeria* species, which can be more challenging to differentiate to species level.

Phylogenetic analysis of the CO1 locus unexpectedly grouped *E. koka* within the same clade as several *Eimeria* described from Australian potoroid marsupials (89–91% sequence homology) (Northover et al., 2019a), which was separate and distinct from the clade of *Eimeria* sequences previously isolated from kiwi. Comparison of *E. koka* to *Eimeria* species described from potoroid marsupials revealed similar morphologic characteristics, such as large size; thick, rough, and/or mamillated oocyst wall; and tan coloration (Barker et al., 1988). While the bootstrap support for monophyly of the *E. koka* -potoroid clade was not high (60.8%), indicating low stability of this clade, it does support *E. koka* as a valid species that has a significant genetic difference to previously described kiwi *Eimeria* species and suggests these species descend from different ancestors. This clustering likely resulted from a lack of relevant closely related reference sequences rather than a (pre)historic host switching event as has been suggested in some other species (Altschul et al., 1994; Zhao et al., 2001). Screening of ITS and larger regions of CO1 should be attempted to gain more resolution on the molecular phylogeny of this new species.

*Eimeria koka* is the first kiwi *Eimeria* species to be described from two distinct host species: North Island brown kiwi and rowi. North Island brown kiwi and rowi are the most closely related species of kiwi (Baker et al., 1995; Burbidge et al., 2003), diverging approximately 1.12 (0.54–2.02) Mya (Weir et al., 2016). They have been geographically isolated in the wild since at least the closure of the last land bridge between the North and South Islands of New Zealand following the last glacial maximum ~ 20,000 years ago (Weir et al., 2016), and there is a lack of evidence for overlap of their wild host ranges, even prior to the separation of these land masses (Shepherd & Lambert, 2008). Due to the short generation time and high fecundity of parasite species (Poulin et al., 2024), genetic divergence in *Eimeria* species that were isolated due to host geographic separation over this time might be expected. For example, another kiwi parasite, the rowi feather louse *Apterygon okarito* (Palma & Price 2004), is morphologically similar to the *Apteryodes* louse found on North Island brown kiwi. Palma suggests this is due to relatedness of their hosts and co-divergent evolution of the parasite with these species of kiwi.

Thus, the presence of a single *Eimeria* species, *E. koka*, confirmed molecularly in both North Island brown kiwi and rowi, despite the geographic isolation of these two flightless kiwi species, seems to suggest a more recent parasite transmission event. This is not the first report of shared *Eimeria* between geographically isolated kiwi species. Coker (2021) observed four species of *Eimeria* isolated from Haast tokoeka faeces that were morphologically synonymous with species previously described from the geographically separate North Island brown kiwi: *E. kiwii, E. apteryxii, E. paopaoii,* and *E .mantellii*. Where parasites evolve in association with one or a few host species, host specificity can break down if new hosts and parasites are brought together out of geographic isolation (Vetterling, 1976; Poulin & Keeney, 2008) and/or when host immunity is reduced, as has been experimentally demonstrated in avian embryos (Fitzgerald, 1970; Nakai et al., 1992). Active conservation efforts in the recovery of all five species of kiwi, which includes the translocation of birds for captive breeding and rearing, and the preservation of genetic diversity, may have inadvertently enabled the transmission of the parasite between these different host populations. In particular, prior to the distinction of rowi as a separate species in 2003, “Haast” tokoeka and rowi were treated as the same species (Tennyson et al., 2003). North Island brown kiwi have been held in South Island captive kiwi facilities (B. Brett, personal communication, September 9, 2024). In addition, at least two or more species of kiwi have historically been held at the same facility through Operation Nest Egg (ONE) (K. McInnes, personal communication, November 14, 2024).

Current management protocols require birds to be clear of coccidia infection, confirmed by faecal oocyst count before translocation, which is sometimes achieved via anticoccidial treatment with toltrazuril (Baycox®, Bayer, Leverkusen, Germany), prior to movement. However, despite biosecurity measures, it seems likely that coccidia, which are shed inconsistently and are extremely environmentally persistent, may be inadvertently transferred between institutions with movements of birds and associated equipment, particularly if anticoccidial treatment is not always efficacious (Sainsbury & Vaughan-Higgins, 2012; Taylor et al., 2019). If so, it might be expected that, over time, kiwi parasite communities, particularly those species with low host-specificity, become homogenous across different captive facilities (Rideout et al., 2017) as has been documented in other endangered hosts that are translocated (Northover et al., 2019b). The ability of *E. koka* (and potentially other species of kiwi *Eimeria*) to host switch might be explained by the genetic similarity of North Island brown kiwi and rowi (Burbidge et al., 2003; Tennyson et al., 2003) and their relatively similar preferred habitat conditions, low population numbers, and their dispersed nature in the environment (Vetterling, 1976). A parasite’s ability to infect multiple closely related hosts would allow them to disperse more readily and persist where host individuals are sparse (Mácová et al., 2018). Further molecular analysis of *Eimeria* species isolated from both tissue and oocyst samples from additional gene regions (ITS, CO1) and across other species of kiwi may provide greater confidence around the shared genetic identity of *E. koka* across North Island brown kiwi and rowi as well as the relatedness of *Eimeria* species across the *Apteryx* genus (Kvičerová et al., 2020).

Previous research has described coccidia infection from the liver, kidney, lung, and spleen of the kiwi in addition to more common enteric infections (Morgan et al., 2012, 2013). Whether extraintestinal infection is more pathogenic in kiwi than enteric forms of the disease is unknown, but it has been suggested in other species that extraintestinal infection can be more challenging to treat effectively (Carpenter et al., 1992). In the present study, the presence of a single genetic identity, synonymous with *E. koka* in infected kidney from the North Island brown kiwi chick pathology case analysed, confirms parasitism of at least one species of kiwi and suggests that, potentially, only a single species of *Eimeria*, *E. koka*, is causing extraintestinal infection. Additionally, in both Morgan (2013) and the present study, asexual stages, and a single morphotype of gametocytes and oocysts distinct from those observed in the intestine (and highly morphological similar to oocysts of *E. koka*) were only observed in the kidney of North Island brown kiwi on histology. This seems to lend further evidence to the possibility that *E. koka* is an extraintestinal species, and that it may be able to complete its entire lifecycle outside of the intestine.

It is possible that the kidney is the preferred sites of infection for *E. koka* Renal coccidiosis is relatively common in avifauna whereas hepatic and other extraintestinal infection is less typical in birds. Hepatic infection, as a primary site of infection, has only been reported in a magpie-lark (*Grallina cyanoleuca*) (Reece, 1989). However, distinct renal *Eimeria* species have been described in geese (*Anser* spp.) (Gajadhar et al., 1982), wild ducks (*Anas* spp.) (Nation & Wobeser, 1977), common loon (*Gavia immer*) (Montgomery et al., 1978), Atlantic puffins (*Fratercula arctica*) (Leighton & Gajadhar, 1986), double-crested cormorants (*Phalacrocorax auritus*) (Yabsley et al., 2002), and herring gulls (*Larus argentatus*) (Gajadhar & Leighton, 1988), amongst others. Alternatively, it is possible that the extraintestinal infections observed in this captive-reared kiwi could be evidence of disseminated coccidiosis, a disease pathology that has been well documented in cranes (*Grus* sp.) (Novilla & Carpenter, 2004) and corncrakes (*Crex crex*) (Serna et al., 2018) within captive-rearing programmes and is thought to be associated with lowered immunity and higher exposure of the parasite to juvenile birds within these programmes.

In contrast, the mixed DNA sequence recovered from the intestinal tissue in this case and the observation of multiple gametocyte and oocyst morphotypes on histology suggest that perhaps multiple kiwi *Eimeria* species infect the intestine, possibly with a similar pattern of infection as is seen in poultry, which have at least five different species of *Eimeria* that infect different portions of the intestine (Jordan et al., 2019). However, in this study, it was not possible to confirm via sequencing whether *E. koka* is one of those intestinal-infecting species. Further study of a larger pool of tissues from kiwi with coccidiosis would be beneficial to describe the endogenous tissue distribution of *E. koka*, and other kiwi *Eimeria* species, and determine their likely primary infection sites in kiwi.

In the present study, morphologically similar oocysts were observed in both North Island brown kiwi and rowi faeces but sequencing of DNA from the oocysts detected in samples from North Island brown kiwi was unsuccessful because they were usually present as a small percentage of a larger mixed species population of coccidia. The prevalence of *E. koka* in faecal samples from all kiwi samples was low. If *E. koka* is confirmed as a renal species, it is most likely that the oocysts are shed via the ureters and that the sample collection method may influence oocyst recovery rate (Tuggle & Crites, 1984; Gajadhar & Leighton, 1988; Page & Haddad, 1995; Greenwald et al., 2024). In kiwi, it is not possible to separate the urine and/or urates from the excrement, and oocysts within the urine/urates could be more prone to losses prior to and during sample collection than faecally excreted oocysts. Further analysis of the urine and/or urate portions of droppings may help to discern whether the sampling process is resulting in these low detection rates or whether there are other factors involved.

This study provides the first description of an extraintestinal species of *Eimeria* infecting kiwi and the first molecularly confirmed record of an *Eimeria* species that infects two distinct kiwi host species. The finding that *E. koka* can infect multiple species of kiwi may provide useful information about potential pathways of transmission of disease when animals are moved between facilities or released to the wild. It also demonstrates the potential utility of molecular methods in confirming parasite species identity in endogenous tissues, particularly in a protected wildlife species such as kiwi where experimental infection or cross-transmission studies to determine intracellular lifecycle stages are not possible. Describing the species that cause infection and having reliable diagnostic tools to identify them are essential steps in being able to characterise the parasite assemblage in different *Apteryx* host species, confirm their distribution within tissues, and link infection with clinical presentations to determine relative pathogenicity. It is recommended that further study of *E. koka* and other species of kiwi *Eimeria* is carried out, to establish each species’ potential pathogenicity in kiwi and to inform methods of management of disease in captive birds.

## Supplementary Information

Below is the link to the electronic supplementary material.

Supplementary file1 (DOCX 6399 KB)

## Data Availability

Sequence data that support the findings of this study have been deposited in GenBank with the accession codes PQ283206 and PQ285481

## Footnotes

1 Massey University School of Veterinary Science’s pathology collection case #51506.
