# Supplementary material for: Morphological and molecular description of a novel species of Eimeria (Apicomplexa) that infects extraintestinal tissues of kiwi (Aves: Apteryx spp.)
Source: Syst Parasitol. 2025 Apr 9;102(3):30. doi: 10.1007/s11230-025-10227-x (PMC11982086; doi:10.1007/s11230-025-10227-x)
Supplement: Supplementary file 1 — Supplementary file1 (DOCX 6399 KB) [file 11230_2025_10227_MOESM1_ESM.docx]

**SUPPLEMENTARY INFORMATION**

Morphological and molecular description of a novel species of *Eimeria* (Apicomplexa) that infects extraintestinal tissues of kiwi (*Apteryx* spp.)

Systematic Parasitology

Authors: Emma Scheltema^1^, Kerri Morgan^2^, Preet Singh^1^, Barbara Adlington^1^, Laryssa Howe^1^

^1^ School of Veterinary Sciences, Massey University, Palmerston North, New Zealand

^2^ Wildbase, Massey University, Palmerston North, New Zealand

Address correspondence to Emma Scheltema, e.scheltema@gmail.com


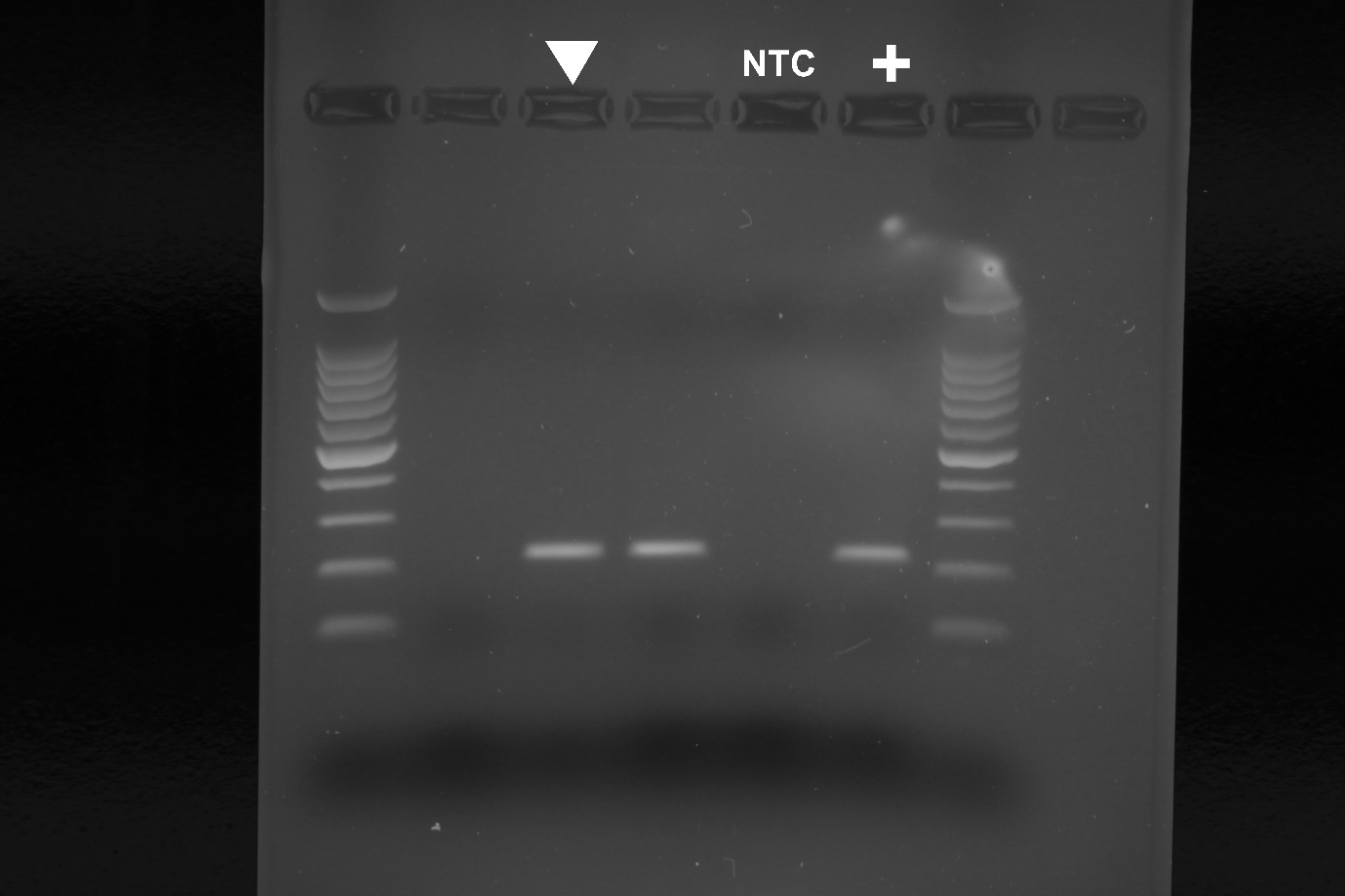


**SM1:** *Eimeria koka* n. sp. faecal DNA PCR product (21021), in lane 2 (arrow head), visualized on 1.5% w/v agarose gel alongside 100bp ladder, was sequenced for this study. A no template control (lane 4) and positive control (lane 5) were also included.


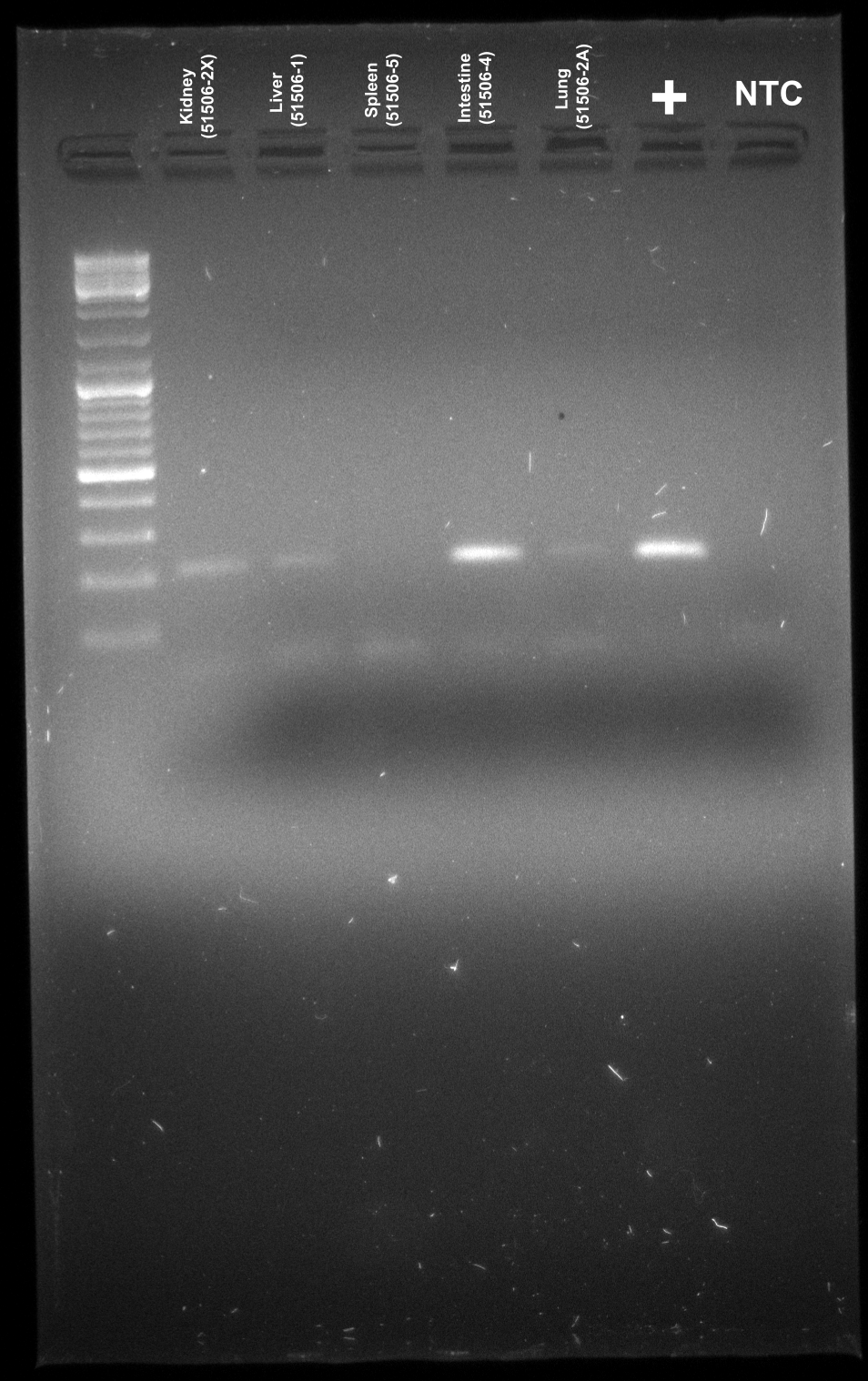


**SM2:** Kiwi (*Apteryx mantelli)* tissue sample (kidney, liver, spleen, intestinal and lung) DNA from a single pathology case (51506), tested with generic *Eimeria* sp. primers (CokerF2/CO1R2), and visualized on 1.5% w/v agarose gel alongside 100bp ladder. A no template control (lane 7) and positive control (lane 6) were also included.
